# Human Group Presence, Group Characteristics, and Group Norms Affect Human-Robot Interaction in Naturalistic Settings

**DOI:** 10.3389/frobt.2019.00048

**Published:** 2019-06-27

**Authors:** Marlena R. Fraune, Selma Šabanović, Takayuki Kanda

**Affiliations:** ^1^Intergroup Human-Robot Interaction Laboratory, Department of Psychology, New Mexico State University, Las Cruces, NM, United States; ^2^R-House, School of Informatics, Computing, and Engineering, Indiana University, Bloomington, IN, United States; ^3^HRI Laboratory, Graduate School of Informatics, Kyoto University, Kyoto, Japan

**Keywords:** human-robot interaction, social robotics, group dynamics, entitativity, group norms, gender

## Abstract

As robots become more prevalent in public spaces, such as museums, malls, and schools, they are coming into increasing contact with groups of people, rather than just individuals. Groups, compared to individuals, can differ in robot acceptance based on the mere presence of a group, group characteristics such as entitativity (i.e., cohesiveness), and group social norms; however, group dynamics are seldom studied in relation to robots in naturalistic settings. To examine how these factors affect human-robot interaction, we observed 2,714 people in a Japanese mall receiving directions from the humanoid robot Robovie. Video and survey responses evaluating the interaction indicate that groups, especially entitative groups, interacted more often, for longer, and more positively with the robot than individuals. Participants also followed the social norms of the groups they were part of; participants who would not be expected to interact with the robot based on their individual characteristics were more likely to interact with it if other members of their group did. These results illustrate the importance of taking into account the presence of a group, group characteristics, and group norms when designing robots for successful interactions in naturalistic settings.

## Introduction

Recent years have seen robots in wider use in public venues and organizational contexts, such as malls, airports, schools, and hospitals. Malls and stores around the world have deployed the humanoid robot Pepper to direct and guide people. In museums, the humanoid NAO guides guests through exhibits (Pitsch et al., 2013). The minimalistic robot Mugbot has been used in nursery schools to read to students and help implement classroom activities (Koike et al., 2009). Along with being used by individuals and families, the socially assistive robot Paro has also been placed in common areas of nursing institutions, where residents can interact with it when and how they like (Wada and Shibata, 2007; Chang et al., 2014).

When one person interacts with a public robot, they often draw other people to interact with it (Weiss et al., 2008; Fraune et al., 2015). Therefore, in public spaces such as those mentioned, robots interact with groups more often than with individual humans (Kanda et al., 2004; Sabanovic et al., 2006). However, such group interaction is seldom studied.

Group interaction introduces new factors that can affect and should be studied in HRI, such as characteristics of the human group (Sabanovic et al., 2006; Johansson and Skantze, 2015). Researchers have also begun to address solutions to group technical problems, such as tracking multiple people in group configurations (Holthaus et al., 2011; Taylor and Riek, 2016; Tseng et al., 2016) or switching attention between multiple people (Bennewitz et al., 2005). However, there are many open questions as to how varied group social dynamics in HRI should be addressed. For example, how should a robot respond when group members show it off to others (Sabanovic et al., 2006) or children debate over who the English-tutor robot liked more (Kanda et al., 2004)?—factors that do not arise in one-on-one interaction. For successful group interaction, it is critical to understand how social group dynamics change and affect perceptions of and behaviors toward robots.

Beyond the mere presence of groups, researchers have found that group behavior is affected both by relational characteristics of group members (e.g., family, coworker), and norms based on individual characteristics of group members (e.g., gender, age; Zanlungo et al., 2017). Although previous studies have also placed robots in public situations where they interact with multiple humans (Al Moubayed et al., 2012; Foster et al., 2012; Gomez et al., 2012; Johansson et al., 2013; Pereira et al., 2014), studies are only beginning to examine the group dynamics of the interaction (e.g., Admoni et al., 2013; Jung et al., 2015; Fraune et al., 2017a; Alves-Oliveira et al., 2019).

**Figure 1:**
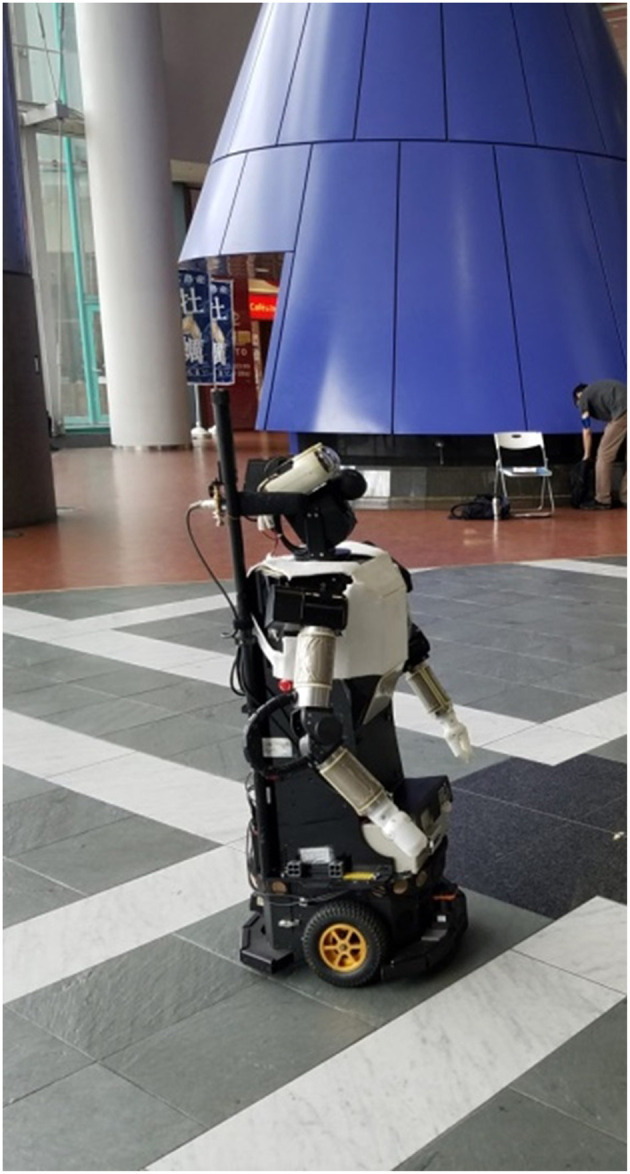
The humanoid robot, Robovie, used in this study.

In this research, we test how the presence of a group, group characteristics, and group norms relate to people's behavior toward a guide robot in a public mall (see Figure 1). We use behavioral and survey measures to answer our questions. Then, we discuss how these variables can be implemented in future robots to enhance interactions with humans.

## Background

### Groups Increase Following Group Goals

When people interact in groups, as opposed to individually, their motivation, and goals shift to be more similar to the group's goals (Reicher et al., 1995). For example, people in a group for a particular political ideology hold that group's ideology and goals more strongly when in that group or thinking of that group than when in a sewing or sports group. This even occurs when people are arbitrarily assigned to groups and had no interaction or common goals with them previously (i.e., minimal groups paradigm; Tajfel et al., 1971). In addition, the goals depend on the interaction context (Sherif, 1936; Gergen et al., 1973; Johnson and Downing, 1979; Fraune et al., 2019; e.g., competitive, collaborative).

When the group's goals are for competition, the discontinuity effect occurs—that is, groups are more aggressive and competitive than individuals (Sherif, 1936; Wildschut et al., 2002, 2003, 2007; Meier and Hinsz, 2004; Wildschut and Insko, 2007; Nawata and Yamaguchi, 2011; Insko et al., 2013). Conversely, when the group's goals are non-competitive or co-operative (e.g., groups must work together to accomplish a shared goal), separate groups co-operate with each other, potentially even combining groups or re-categorizing into one group to complete a shared goal (Sherif, 1936; Anastasio et al., 1997; Gaertner et al., 2000).

This pattern also occurs in HRI. In competitive situations, human groups competed more than individuals against robots (Chang et al., 2012; Fraune et al., 2019). In naturalistic environments, groups of unaccompanied children were more aggressive toward robots than individual children (Brscić et al., 2015). Conversely, interacting with robots in a learning context, groups of children were not more negative toward the robots (Leite et al., 2015). In naturalistic settings, groups of humans, rather than individuals, were more likely to stop to interact with robots (Weiss et al., 2008; Fraune et al., 2015).

In this study, a humanoid robot gave directions in a mall and actively sought to help participants. In this context, we expect that the typical participant goal while interacting with the robot will be to explore or seek guidance, and that people would be positive about the experience. The group should hold this goal more strongly than individuals. Therefore, we hypothesize:

**H1. Presence of a group**. Groups will be more positive and willing to interact with a robot than individuals, as measured in positive survey responses and duration of interaction with the robot.

### Entitativity Increases Following Group Goals

Group entitativity magnifies certain characteristics of groups. Entitativity is defined as group cohesiveness, which includes group members sharing similar static traits (e.g., background, appearance, that are unlikely to change) and dynamic traits (e.g., goals, and outcomes, that may change frequently; Campbell, 1958). Entitativity increases group identification (Castano et al., 2003), and norms for behavior Lickel et al., 2000, which motivate members to achieve the goals of the group. The more entitative a group is, the more the group's behavior aligns with its goal (Gergen et al., 1973; Insko et al., 1988, 2013).

In competitive contexts, group entitativity magnifies the discontinuity effect, increasing competition, and aggression (Gaertner and Schopler, 1998; Insko et al., 2013) across cultures (Kumagai and Ohbuchi, 2009). In co-operative or positive contexts, group entitativity increases positivity (Gergen et al., 1973; Johnson and Downing, 1979). For example, when participants were inserted into cohesive groups, context cues of group harshness (e.g., KKK) influenced behavior to be more harsh than cues of group kindness (Gergen et al., 1973; Johnson and Downing, 1979; e.g., vs. nurse or hippie). In the case of a mall guidance robot, group entitativity should increase participants' exploratory and positive manner toward the robot. We hypothesize:

**H2.Group characteristics**. Entitative groups will be more positive and willing to interact with the robot than Diverse groups.

Although the effects of human group entitativity in naturally-occurring HRI have not yet been examined, robots can detect factors of entitativity. Robots have been capable of accurately predicting child friend groups based on proximity (Kanda et al., 2007) and adult groups based on how they interacted with each other (Giuliani et al., 2013). Therefore, if group membership and entitativity is useful in determining appropriate robot behavior, practitioners could develop algorithms to detect these patterns in naturalistic settings.

#### Group Type as a Natural Indicator of Group Entitativity

Group entitativity has been shown to vary across different types of groups. Entitativity is typically high in intimacy groups (e.g., family, friends), medium in task groups (e.g., coworkers), and low in loose associations (e.g., people standing in line; Lickel et al., 2000). Thus, in this study, we hypothesize that:

**H2a**.Family and friends will be more positive toward and interact more with the robot than colleagues because…
**H2ai**.Family and Friend groups will be more entitative than Other groups (e.g., coworkers) as measured in the survey.

A second likelihood is that intimacy groups will more commonly share a group goal of leisure and exploration in the mall, whereas task groups will share more group goals of getting a job done. We did not measure this, and future studies should examine goals specific to groups; however, in this study, we do have multiple measures of group entitativity.

Whereas, prior research investigated entitative groups that were created in the lab, this study examines naturally-occurring groups in a public space. This is critical because artificially-created lab groups may be loose associations or even task groups centered on a task, but are typically not intimacy groups. This is the first study to examine intimacy groups in intergroup HRI.

#### Gender as a Natural Indicator of Group Type in Japan

Literature in social psychology indicates that gender differences in behaviors and attitudes occur across cultures (Costa et al., 2001). Gender differences, while small on their own (e.g., explaining 5% of the variance aggression; Hyde, 1984), are increased (Hyde, 1984; Eagly and Wood, 1999) by differences in the social roles that people of each gender occupy (Rosario et al., 1988). In particular, in Japan (where we conduct this study), gender strongly correlates with occupation. That is, in Japan, males are more likely to be business people and managers, and females are more likely to be homemakers (Wright et al., 1995; Steinberg and Nakane, 2012) even in 2018, females made up only 43% of the labor force in Japan (“Labor Force., female (% of total labor force),” 2018). These gender differences are likely to account for differences in HRI in naturalistic settings. Therefore, although gender effects in HRI are mixed (Siino and Hinds, 2005; Schermerhorn et al., 2008; Siegel et al., 2009; Eyssel et al., 2012), we expect that in this situation, gender effects will be primarily driven by females being part of family and friend groups and therefore more entitative (argued above). Relatedly, past research indicates that females expected robots to be helpful in their personal lives (like family and friends), whereas males expected robots to be helpful in their work (Wang, 2014). This leads us to hypothesize:

**H2b**.Females will interact with the robot for longer and rate it more positively than males, especially coworkers. Because…
**H2bi**.Females will be in more family and friend groups (as measured by reported group type, video coded group type, and more children with them), and males in more coworker groups. Additionally, because…
**H2bii**.Females will rate their groups as more entitative (because they are more likely to be with family and friend groups).

### Groups Influence People to Follow Group Norms

Group norms set expectations for typical behavior (Cialdini, 2007; Smith et al., 2007; Goldstein et al., 2008; Burger and Shelton, 2011). For example, how people respond to death and whether they take the stairs or elevator (Burger and Shelton, 2011) depends on ingroup norms (Goldstein et al., 2008) that are embedded in their culture or explicitly stated (Cialdini, 2007). In unfamiliar situations in which people do not otherwise know how to act, they are especially likely to follow norms they observe (Smith et al., 2007).

Within HRI, because robots are unfamiliar, people often look to norms when interacting with them (Lee et al., 2010; Chang et al., 2014). In the present study, seeing other people interact with the robot creates a norm of interacting and may induce more people to interact with it. Further, creating a norm that is more relevant to participants may increase following the norm (Goldstein et al., 2008). For example, participants with their family may be more likely to interact with the robot if they see their family member interact with it than if they see a group of friends interact. We hypothesize:

**H3**.Participants will be more likely to interact with the robot if they previously see someone from their group interact with the robot.

According to H2B, males will be least likely to interact with the robot. We hypothesize group norms will change this behavior as follows:

**H3a**.Males will interact more with the robot if others in their group interact than if others do not interact with it.
**H3b**.Males will interact more with the robot if there are more females in their group.

### Overview

In the study, we examine how presence of groups, group characteristics, and group norms influence people's behavior toward robots. We do so by placing a humanoid robot in a public mall in Japan and using survey and video data to determine group and individual characteristics and the valence of participant responses toward the robot. The results will help practitioners account for and make adjustments to robot designs to enhance interaction depending on the context of interaction and characteristics of human groups involved in the interaction.

## Methods

This study was approved by the Institutional Review Board (IRB) at Indiana University (Protocol code 1606171019). Informed consent was not required for video recordings, as interactions occurred in a public setting, but a large sign was placed in the area indicating that it was being recorded. Verbal consent was obtained for survey participants.

### Procedure

**Figure 2:**
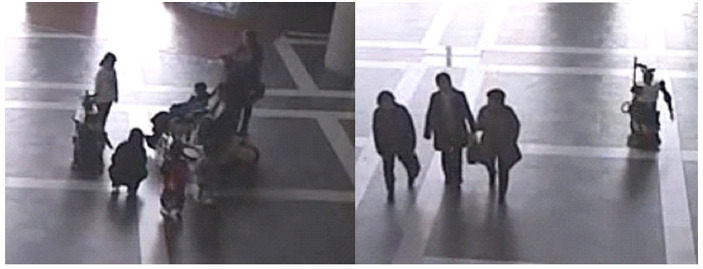
Robovie robot in the ATC Mall with Age-Diverse **(Left)** and Age-Similar **(Right)** groups.

In a large open area in the Asian Trade Center (ATC) mall in Osaka, Japan, we placed the humanoid robot Robovie (Figure 2). Robovie remained stationary and waited for people to approach. When someone was ~1 m in front of or to the side of Robovie, the robot detected and turned toward them.

The robot introduced itself and asked where visitors would like to go. It directed visitors to that location by turning, pointing, and describing the path they should take. Robovie allowed participants to ask for directions to multiple locations. As participants began to walk away, Robovie said, “Bye-bye.” The entire time, Robovie switched between making eye contact with participants and looking toward where it was pointing to share joint attention in the direction participants should travel.

Between two and three researchers stood spread out on the outskirts of the open mall area and intercepted participants who had interacted with the robot long enough for Robovie to speak at least three sentences. If there were multiple people in a group, the researchers asked everyone to take a survey on a clipboard. The researchers requested that participants take the survey far from the robot so as to not interrupt anyone else's interactions with Robovie.

Video cameras recorded interactions with Robovie with a wide angle from above.

The experiment took place over the course of 21 days between October 2016 and February 2017. Each day, the robot was placed in the mall for ~3 h at a time. For the purpose of this study, ~20% of videos, randomly chosen, were coded for a total of approximately 12 h of analyzed video. Surveys were included in the analysis regardless of whether or not they overlapped with video that was coded.

### The Humanoid Robot Robovie

Robovie (Figure 2) has two arms (each with four degrees of freedom [DOF]), a head (3 DOF), two eyes (each with 2 DOF), a mobile platform (two driving wheels and one free wheel), 10 tactile sensors, an omnidirectional vision sensor, two microphones to listen to human voices, and two laser rangefinders for detecting obstacles. The eyes have a pan-tilt mechanism with direct-drive motors, and they are used for stereo vision and gaze control.

Although Robovie can function fully autonomously, for the purposes of this study we controlled certain aspects of Robovie's behavior via a wireless local area network (IEEE 802.11a LAN), employing a Wizard of Oz (WoZ) technique. We employed this technique because in the loud mall environment, the robot has difficulty parsing human speech. In this study, Robovie's gaze and direction-giving behaviors were autonomous, but a Japanese researcher typed the locations participants wanted to go to into a computer so the robot could respond accordingly. From there, the robot autonomously directed them on how to get there.

### Participants

Participants were people in the ATC Mall in Japan. Survey and video demographics are summarized in Tables 1, 2 below. In the video, participants were included if they were visible enough in the video frame that demographic information could be collected about them. Thus, a total of 2,714 participants were coded in the video (some of whom interacted with the robot) and 375 participants took the survey. Seventy-eight participants were both coded in the video and took the survey.

**Table 1:** Survey demographics.

	**Demographics**		**Experience with…**		**Group type**			
	**Age (*M*)**	**Gender (%)**	Computer experience (*M*)^*^	Robot experience (*M*)^**^	**Alone (*N*)**	**Family (*N*)**	**Friend (*N*)**	**Coworker (*N*)**
*M/N/%*	37.75	50.20 Female	2.15	3.00	12	219	60	43
Std. Deviation	14.22		1.53	5.17				

* *Scale: from 1 (Novice) to 5 (Programmer)*.

** *Scale: 1 (None) to 5 (Build robots)*.

**Table 2:** Video demographics.

	**Age**					**Group type**			
	**Older adults**	**Adult**	**Teenager**	**Child**	**Baby**	**Age-diverse**	**Age-similar**	**Business-dressed**	**Business-young**
Frequency	60	2,409	50	145	52	535	516	106	20
Percent	2.2	88.6	1.8	5.3	1.9	19.7	19	3.9	0.7

### Measures

#### Survey

The survey took ~2 min (see Appendices A,B for full survey in English and Japanese, respectively). Questions asked participants to report on:

- Group Characteristics
  ° Group size (number of members; free response)
  ° Group type (family, friend, coworkers, acquaintances, alone)
  ° Entitativity (i.e., cohesiveness with group, similarity of members in the group) was rated on a Likert scale from 1 (Strongly Disagree) to 7 (Strongly Agree) for human groups (Cronbach's α = 0.826) and humans with the robot (α = 0.824)
- Participant characteristics
  ° Experience with computers (novice, comfortable for simple tasks, comfortable for moderately complex tasks, comfortable programming) and robots (seen none, in media/TV, interacted with, own one or more, work with/build)
  ° Year born (free response)
  ° Gender (free response)
- Ratings of Robot
  ° Perceptions of the robot on a Likert scale was rated on a 9-point semantic differential Likert scale (from 0 to 8, i.e., negative-positive, scary-friendly, mean-kind, useless-useful, stupid-smart, non-social-social, machinelike-humanlike; Fraune et al., 2015)
  ° Willingness to interact (enjoyment of the interaction, would interact with the robot in the future, would recommend for others to interact with the robot) was rated on a 9-point Likert scale from 0 (Definitely not) to 8 (Definitely yes).

#### Video

Video data were coded by four independent coders using ELAN. They were trained to code the data in the same way using 60 min of video from the study. They did this in 10 min segments, coding independently, and then meeting to discuss differences in codes. This process was continued until agreement was 80% or higher. Then video segments were assigned to the coders to work on independently based on the times they were available.

Approximately 20% of videos were coded by two of the four coders who did not know that someone else was coding the same video. Finally, we calculated percent agreement across all videos. We calculated percent agreement because other measures of interrater reliability were not feasible for the thousands of participants and coding method we used in this study. Due to the large number of participants, individual coders often gave different participant numbers to each participant, but generally coded them similarly (e.g., Coder 1 may have seen someone walk in from the left side of the camera first and labeled that person Participant 1, while Coder 2 saw a group walk in from the right and labeled them Participants 1–4, then labeled the participant from the left as Participant 5, but looking closely, Coder 1's Participant 1 and Coder 2's Participant 5 match up in terms of coded gender, time spent looking at the robot, etc.).

For deciding which codes to include in the data analysis for video sections that were coded by multiple coders, codes were taken from coders who had the highest interrater reliability across videos.

Videos were coded for the variables described in Table 3. Percent agreement in specific video segments ranged from 60 to 100%, but was averaged for overall percent agreement, included in Table 3.

**Table 3:** Description of video coding scheme and operational definitions of variables.

**Variable**	**Percent agreement**	**Code**	**Operational definition**
Interaction with robot	82.31%	No	
		Yes (duration)	Participants came close enough to Robovie that it would begin talking to them
Duration of interaction (seconds)	89.86%		Began when participants entered the interaction space with the robot and ending when participants left the space and had stopped looking at the robot.
Social gesture toward robots		No	
	76.94%	Yes	Participants made social gestures toward the robot (e.g., After interacting with the robot, participants turned back and waved).
Age	84.26%	Older adults	Looked to be approximately older than 55 years (e.g., moved more slowly).
		Adults	Looked to be between 18 and 55 years (e.g., tall, medium pace).
		Teenagers	Looked to be between 13 and 18 (e.g., short, often with adults or wearing school uniforms).
		Children	Looked younger than 13, but could walk on their own (e.g., were shorter, more likely to run, and often with adults or older adults).
		Babies	Could not walk on their own (e.g., in a stroller or carried the entire time on camera).
		*Three participants were excluded on analyses of age because their age is impossible to estimate given the camera angle.	
Gender	Matched survey 98.7% of the time	Male	Appeared to be male.
		Female	Appeared to be female.
		Undefined	Used when gender could not be determined, in particular with babies.
Gender Proportion	Calculated by computer based on the above codes.	All Male	
		Mostly Male	Between 50.1% male and 99.9% male
		50/50	
		Mostly Female	Between 50.1% female and 99.9% female
		All Female
Group	75.75%	No	Participant was alone
		Yes (divided into categories below; Figure 2)	Participants walked in close formation with each other and spoke with each other during their time on the camera. The divisions below are mutually exclusive.
		Age-Diverse	Groups with diverse ages
		Age-Similar	Groups with similarly-aged participants
		Business-Dressed	Groups of adults dressed in suits or other business wear (Martin and Chaney, 2012). If participants fit the criteria for business-dressed, they were coded in this category rather than age-diverse or age-similar.
		Business-Young	Groups of children or young adults dressed in school uniform. If participants fit the criteria for business-young, they were coded in this category rather than age-diverse, age-similar, or business-dressed.
		*Data were excluded from Group Type analyses in 36 cases in which the group type could not be determined (e.g., because some group members were partially excluded from the camera frame resulting in being unable to tell what type of group it was).	
Group Size	76.36%	Participants who were in a group were coded to be in a group with a certain number of participants–one (alone) to six (the maximum group size coded).	
Seen Previous Interaction	Calculated by computer based on the above codes.	No	
		Yes	Participants were considered to have seen a previous interaction if another participant was coded as having interacted with the robot <10 s before the current participant. This included if the participant saw another person approach the robot and the participant approached the robot while the other person was still interacting with it.
Group of Previous Interaction	Calculated by computer based on the above codes.	No Previous	No interaction occurred 10 s before participants interacted
		Different Group	A group that was not the participant's group interacted within 10 s of the participant interacting
		Different Group and Individual	A group and individual, both not of the participant's group, interacted with the robot 10 s before the participant interacted
		Own Group	The participant's own group interaction with the robot 10 s or less before the participant appeared on screen

## Results

Data were analyzed in SPSS 24. *P*-values of < 0.05 were considered statistically significant. When we conducted multiple tests, we used Bonferroni corrections. To promote open science, deidentified video and survey data can be found at https://osf.io/ew8ta/?view_only=df9ef0f0919b48afb64a773ffa0251ac.

First, we examine if survey and video measures cohere. Next, we test corollary hypotheses (H2ai, H2bi, H2bii). Finally, we test the main hypotheses about main effects of group and gender (H1, H2a, H2b), linear regression of entitativity (H2), and effects of norms (H3, H3a, H3b).

### Survey and Video Measures of Interaction and Gender Were Consistent. Measures of Group Type Differed

#### Interaction

Everyone who completed the survey had interacted with the robot as coded in the video. Of 2,714 video-coded participants who walked through the video, ~15% interacted with the robot. Participants who interacted with the robot did so for an average of 47.8 s and a median of 37.3 s. The maximum time participants interacted with the robot was 289.3 s or 4 min and 49.3 s. A normality test revealed that skewness of interaction time was at an acceptable level (1.88).

#### Gender

Of 78 surveys (32 female, 46 male) that overlapped with coded video, one self-identified male was coded as female for a 98.7% accuracy rate for video coding. This false code was changed to match the survey data. In the video, there were 1,655 Males, 968 Females, and 91 Undefined either because they were too young to tell their gender or the coder did not have a good view of their face. In this study, Undefined were excluded from gender analyses because all but three Undefined were children.

#### Groups

Groups had an average (mean) of 2.69 members (*SD* = 0.95), with a minimum of two and maximum of six. In surveys, some participants indicated both Family and Friend as group types. For these, they were recoded as Family groups. Video coding and survey responses related to each other as described: Families and coworkers were typically coded as Age-Diverse groups (more than 50%) and sometimes as Age-Similar (about 25%). Coworkers were coded as Business-Dressed almost 50% of the time. On some occasions, loose acquaintances and strangers were coded as Age-Diverse or-Similar groups. Those who were alone were coded as Alone 100% of the time (Table 4).

**Table 4:** Comparing video coding with survey description of group type.

	**Survey description of group**						**Total**	
	**Family**	**Friend**	**Coworker**	**Loose acquaintance**	**Strangers**	**Alone**		
Video-coded group type	Age-diverse	31 (69%)	7 (58%)	2 (22%)	1 (50%)	1 (50%)	0	42 (55%)
	Age-similar	12 (27%)	3 (25%)	2 (22%)	1 (50%)	1 (50%)	0	19 (25%)
	Business-dressed	0	0	4 (44%)	0	0	0	4 (5%)
	Business-young	2 (4%)	2 (17%)	0	0	0	0	4 (5%)
	Alone	0	0	1 (11%)	0	0	7 (100%)	8 (10%)
Total	45	12	9	2	2	7	77	

*738 survey description of group*.

### Females Were in More Family and Friend Groups, and Males in More Coworker Groups (H2bi)

In this section, we included the variable 2 (Gender: Male, Female) for most of the tests.

#### Females Were in More Family and Friend, and Males in More Coworker Groups, According to Survey Responses

**Figure 3:**
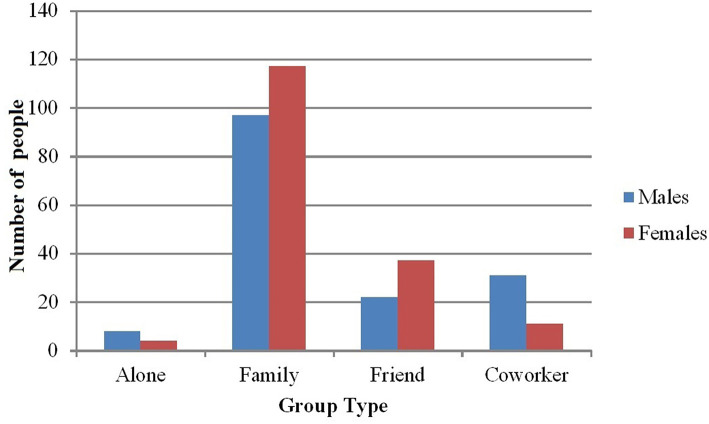
Number of males and females in different group types according to survey ratings.

We ran a chi-squared test on 2 (Gender) × 4 (Group Type: Alone, Family, Friend, Coworker) reported in the survey. Loose Acquaintances, Stranger, and Other were excluded because they violated the expectation of having at least five counts per cell and they did not fit logically into the Alone, Family, Friend, or Coworker groups. Results indicated a significant relationship between Gender and Group Type (*X*^2^(3, *N* = 327) = 16.19, *p* = 0.001) such that Males were more likely to be in groups with Coworkers (*Adjusted Standardized Residual*; *ASR* = 3.5) and slightly less likely to be in groups with Friends (*ASR* = −1.9) than Females (Figure 3).

#### Females Were Less Likely to be Alone or in Business-Dressed Groups Than Males, According to Video Responses

**Figure 4:**
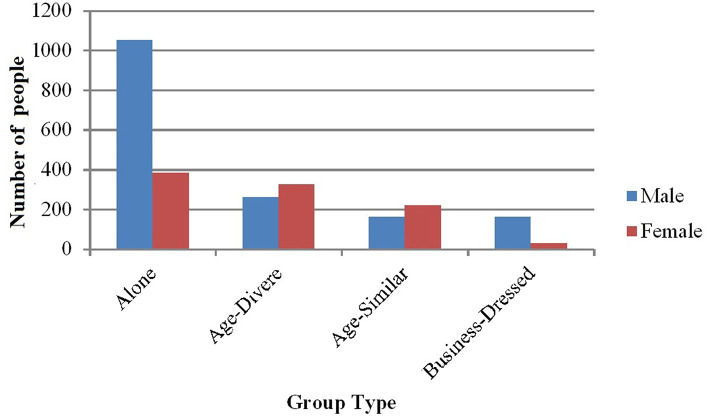
Number of males and females in different group types as observed in the video.

We ran a similar chi-squared test on video data: 2 (Gender) × 4 (Group Type: Alone, Age-Diverse, Age-Similar, Business-Dressed). We excluded Student Groups because it violated the expectation of having at least five counts per cell. Results indicated a significant interaction effect of Gender and Group Type (*X*^2^(3, *N* = 2,605) = 259.46, *p* < 0.001) such that Males were much more likely to be Alone (*ASR* = 11.9) or in groups of Business-Dressed (*ASR* = 6.5) than in groups of Age-Diverse (*ASR* = −10.7) or Age-Similar (*ASR* = −9.0) compared to Females (Figure 4).

#### Females Were More Likely to Have Children in Their Group Than Males, Suggesting Family Ties

**Figure 5:**
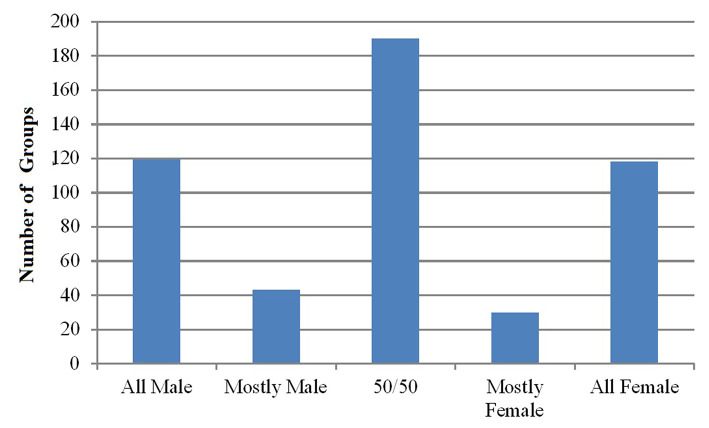
Gender distribution in video-coded groups.

Gender proportion in groups, as coded on video, are reported in Figure 5. A 5 (Age: Older Adult, Adult, Teenager, Child, Baby) × 5 (Gender Proportion: All Male, Mostly Male, 50–50, Mostly Female, All Female) chi-squared test showed that Age interacted with Gender Proportion in Group (*X*^2^(16, *N* = 1,273) = 125.66, *p* < 0.001). Primary-female groups contained more teenagers, children, and babies, but fewer adults than expected. Primary-male groups included more adults, teenagers, and fewer older adults and children than expected. 50/50 groups contained more elderly and adults and fewer teens and children (Table 5).

**Table 5:** Percent of participants, divided by Gender Proportion and Age (ASR). Negative ASR values indicate that the percent was lower than expected.

	**Older adult**	**Adult**	**Teenager**	**Child**	**Baby**
All male	1.01 (−2.1)	84.80 (3.5)	6.76 (2.8)	4.39 (−4.3)	3.04 (−1.3)
Mostly male	0.72 (−1.6)	69.78 (−2.3)	2.16 (−1.2)	24.46 (5.2)	2.88 (−0.9)
50/50	4.41 (2.4)	81.90 (2.7)	0.46 (−4.6)	8.58 (−2.2)	4.64 (0.3)
Mostly female	1.00 (−1.1)	68.00 (−2.4)	0.00 (−2.1)	28.00 (5.5)	3.00 (−0.7)
All female	3.91 (1.3)	70.68 (−3.3)	8.47 (4.6)	10.42 (−0.6)	6.51 (2.1)

### Families Were More Entitative Than Friends and Coworkers (H2ai), and for Friend Groups, Females Rated Groups as More Entitative Than Males (H2bii)

An ANOVA revealed a main effect of Group Type (*F*(2, 298) = 3.45, *p* = 0.033, $n_{\text{p}}^{2}$ = 0.007) such that Families rated themselves as more Cohesive than Friend groups did and as more similar (*F*(2, 290) = 3.90, *p* = 0.021, $n_{\text{p}}^{2}$ = 0.026) than Coworkers (*p* = 0.017). An interaction effect between Group Type and Gender (*F*(2, 298) = 3.73, *p* = 0.025, $n_{\text{p}}^{2}$ = 0.024) indicated that Female Friends rated their groups as more cohesive than Male Friends, but otherwise Males and Females rated their group similarly in relation to their cohesion.

### Groups (H1), Especially Age-Diverse and Age-Similar (H2a), and Females (H2b) Were Typically More Positive Toward Interaction With the Robot

In this section, we included the variable 2 (Gender: Male, Female) for most of the tests. Categories were excluded in cases having 12 or fewer participants.

**Females were more positive toward the robot than males according to** ***survey responses*** **(H2b), but there was no main effect of group (H1) or group type (H2a)**. We ran a series of 2 (Gender) × 3 (Family, Friend, Coworker) ANOVAs on survey responses. Main effects of Gender indicated that Females rated more enjoyment (*F*(2, 306) = 5.59, *p* = 0.019, $n_{\text{p}}^{2}$ = 0.018) and usefulness in the future (*F*(2, 309) = 4.55, *p* = 0.030, $n_{\text{p}}^{2}$ = 0.015) from the robot than males. No main effects of Group Type were found. An interaction effect revealed that Females rated the robot as less smart than Male when in Coworkers groups, but Females rated the robot as smarter than Males did in Family and Friend groups (*F*(2, 306) = 4.55, *p* = 0.011, $n_{\text{p}}^{2}$ = 0.029).

**Groups (H1), especially Age-Diverse and Age-Similar (H2a) were** ***more likely to interact* with the robot than Alone participants. Females were more likely to interact than males (H2b)**. We ran a 2 (Gender) × 4 (Group Type: Alone, Age-Diverse, Age-Similar, Business-Dressed) × 2 (Interaction: Yes, No) Chi squared test on whether or not participants interacted with the robot. There were statistically significant differences (X^2^(3, *N* = 2,605) = 339.94, *p* < 0.001; see Table 6). Groups of Age-Diverse or Age-Similar participants were more likely, and Alone participants were less likely, to interact than expected. When divided by Gender, the same was true of Females, but for Males, only Age-Diverse (not Age-Similar) were more likely, and those who were Alone were less likely, to interact than expected. Further, Females were more likely to interact than Males (X^2^(1, *N* = 2,605) = 36.68, *p* < 0.001).

**Table 6:** Percent of participants who interacted with the robot (ASR), divided by gender and group type.

	**Alone**	**Age-diverse**	**Age-similar**	**Business-dressed**	**Total**
Male	3.42 (−13.3)	35.88 (13.9)	20.12 (3.9)	12.12 (0.4)	11.14 (−6.1)
Female	4.95 (−9.4)	39.33 (11.0)	17.27 (−1.1)	13.33 (−0.9)	19.75 (6.1)
Total	3.83 (−16.9)	37.80 (18.5)	18.49 (2.5)	12.31 (−0.8)	

**Figure 6:**
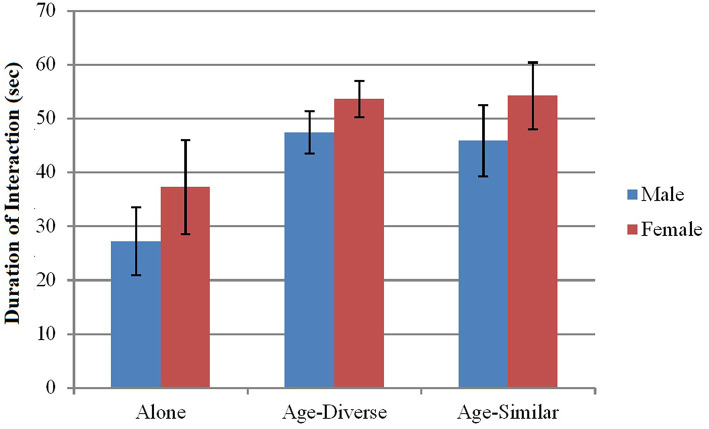
Group type and gender on duration of interaction. Error bars indicate standard error.

**Groups (H1), especially Age-Diverse and Age-Similar (H2a)** ***interacted for longer* with the robot than Alone participants. Females were** ***not* more likely to interact for longer than males (H2b)**. Excluding participants who did not interact with the robot, a 2 (Gender) × 3 (Group Type: Alone, Age-Diverse, Age-Similar) ANOVA indicated a main effect of Group Type (*F*(2, 343) = 4.85, *p* = 0.008, $n_{\text{p}}^{2}$ = 0.028) such that participants who were Alone interacted for a shorter time with the robot than those in Age-Diverse (*p* = 0.001) and Age-Similar groups (*p* = 0.013). There were no differences among those who were in groups. No main effects of Gender or interaction effects were found (Figure 6).

**Group (H1) and Group Type (H2a) did not affect** ***social gestures* toward the robot. Females were more likely to socially gesture than males (H2b)**. We ran a 2 (Gender) × 3 (Group Type: Alone, Age-Diverse, Age-Similar) chi-squared test on whether or not participants made social gestures toward the robot. There was a main effect of Gender (X^2^(1, *N* = 2,425) = 17.04, *p* < 0.001) such that Females were more likely to make social gestures toward the robot than Males. No main effect of Group Type or interaction effects occurred (Table 7).

**Table 7:** Percent of participants who socially gestured toward the robot as divided by Gender and Group Type [Adjusted Standardized Residual (ARS)].

	**Alone**	**Age-diverse**	**Age-similar**	**Total**
Male	0.57 (−10.3)	12.98 (8.5)	10.37 (4.6)	3.86
Female	1.30 (−6.3)	16.46 (7.1)	6.82 (−0.7)	7.94
Total	0.77 (−12.3)	14.92 (11.7)	8.33 (2.7)	5.44

### High-Entitative Groups Were Slightly More Positive Toward the Robot Than Low-Entitative Groups (H2)

Linear regression indicated no relation between perceived ingroup entitativity and ratings of the robot (*p*s > 0.050), except that participants who rated their group as highly entitative were more likely to recommend for others to use the robot (*F*(1,98) = 6.91, *p* = 0.010; *B* = 0.323; *R* = 0.257). The equation was: Likeliness to recommend = 2.89 + 0.323 ^*^ (Human group entitativity).

### Norms of Interaction Affected Participants (H3), Especially Males (H3a, H3b)

Survey responses related to gender ratio were too few (*N* = 69), so for tests of norms we examined only behavior.

**Figure 7:**
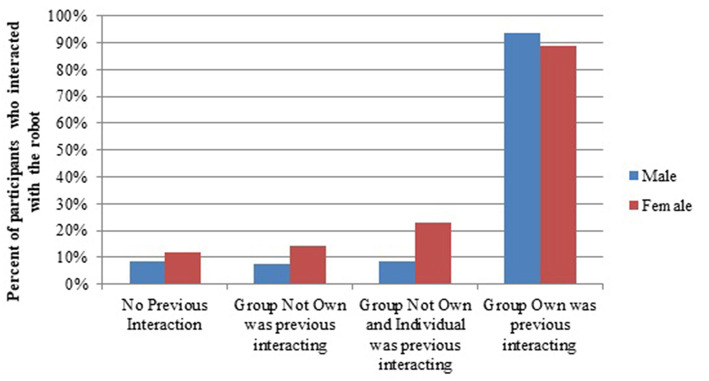
Interaction with the robot based on previous exposure to interactions with it.

**Participants (H3), especially males (H3a) were more likely to** ***interact* with the robot if others in their group previously interacted**.. Overall, a 2 (Gender: Male, Female) × 4 (Seen Previous Interaction: No Previous, Different Group, Different Group and Individual, Own Group) × 2 (Interaction: Yes, No) chi-squared test revealed that participants were more likely to interact than expected with the robot if they saw Own Group (*ASR* = 27.3) interact or currently interacting with the robot, and less likely to interact than expected if they saw No Previous (*ASR* = −10.0) or Different Group (*ASR* = −4.1) interacting (X^2^(3, *N* = 2,466) = 745.96, *p* < 0.001; Figure 7), regardless of gender. There was an interaction effect between Gender and Seen Previous (X^2^(1, *N* = 2,466) = 36.84, *p* < 0.001). Females were more likely to interact than expected compared to Males when they saw No Previous (*ASR* = 2.2, *p* = 0.031) interaction, Different Group (*ASR* = 2.7, *p* = 0.007), or Own Group, and Individual (*ASR* = 2.2, *p* = 0.025). However, there was no significant difference in Male and Female interaction with the robot when Own Group had previously interacted (*ASR* = −1.0, *p* = 0.315).

**Participants (H3) were more likely to** ***socially gesture* toward the robot if others in their group previously interacted. Gender did not affect the relationship (H3a)**. When the same test was run on Gesture (Yes, No), the Expected Count for Own Group Male Gesture was too low (*N* = 2.4). However, the effects were similar across gender. Therefore, Gender was collapsed and a 4 (Seen Previous Interaction: No Previous, Group Not, Different Group and Individual, Own Group) × 2 (Gesture: Yes, No) chi-squared test was run (X^2^(3, *N* = 2,466) = 133.31, *p* < 0.001) indicating that participants gestured at the robot less often than expected when they saw No Previous (*ASR* = −3.9) and more often when they saw Own Group (*ASR* = 11.5) interacting. Because Different Group and Different Group and Individual showed interaction in similar direction, they were combined to find that participants who saw those not in their group interacting were also less likely to make a social gesture toward the robot (*ASR* = −2.5; X^2^(3, *N* = 2,466) = 140.84, *p* < 0.001).

**Figure 8:**
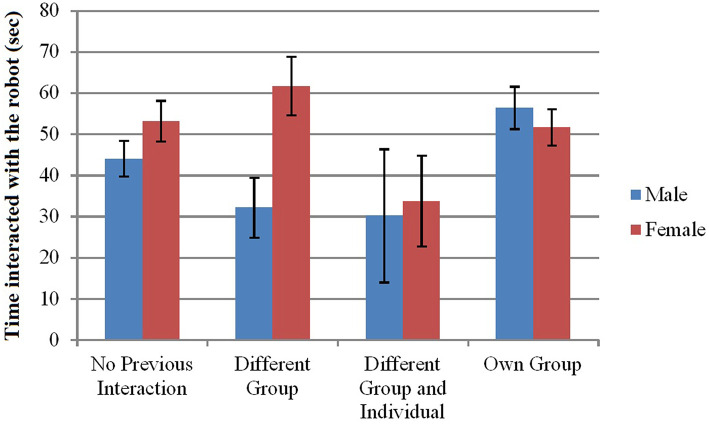
Effect of gender and group type on time interacted with the robot. Error bars indicate standard error.

**Males (H3a)** ***interacted for a longer duration* with the robot if others in their group had previously interacted. The effect was not clear for participants overall (H3)**. A 2 (Gender: Male, Female) × 4 (Seen Previous Interaction: No previous, Different Group, Different Group and Individual, Own Group) ANOVA was run on interaction time for participants who interacted with the robot. There was an interaction effect between Gender and Seen Previous Interaction (*F*(3, 359 = 2.69, *p* = 0.046, $n_{\text{p}}^{2}$ = 0.022) such that Males interacted for less time than Females unless they saw their Own Group interacting (Figure 8).

**Males** ***interacted more* with and made more** ***social gestures* toward the robot if there were more females in their groups (H3b)**. We ran logistic regressions for the effects of Gender and Gender Ratio on behavior relating to the robot (*N* = 1,270). On interaction (Y/N), (Nagelkerke's *R*^2^ = 0.015, *X*^2^(5) = 61.89, *p* < 0.001), only the effect of Gender Ratio was significant (Wald(1) = 9.23, *p* = 0.002, *B* = 0.749) such that the greater percentage female in the group, the more likely participants were to interact with the robot. The same was true of gesture (Nagelkerke's *R*^2^ = 0.015, *X*^2^(5) = 16.67, *p* = 0.011) (Wald(1) = 4.98, *p* = 0.026, *B* = 0.818). When Gender Ratio was used as a covariate in the 2 (Gender) × 3 (Group Type: Age-Diverse, Age-Similar, Business-Dressed), no effects were found.

## Discussion

In this study, participants interacted with a humanoid robot in a mall setting. Participants who interacted with the robot were given the opportunity to complete a survey. Behavior of those who passed through the area was examined by independent video coders with high accuracy. The main findings of the study were twofold: Groups (H1, H2a, H2b), especially entitative groups (H2, H2ai, H2bii) increased following of group goals, and group norms of interaction increased actual interaction (H3). These findings are described in more depth below.

### H1 and H2. Groups, Especially Entitative Groups, Enjoyed the Robot

Groups, especially entitative groups, as compared to individuals, (1) interacted more and for longer with, (2) behaved more socially toward, and (3) were more positive toward a robot in the mall. These results were shown across survey and behavioral measures, supporting H1, H2a, and H2b. These findings indicate that in a naturalistic setting, groups had more positive interaction with the robot than individuals—at least in the positive and friendly mall environment. They also contribute the novel information that the entitativity of pre-existing participant groups can positively affect subjective and behavioral responses toward a robot in a naturalistic setting. These results are useful for HRI because they show that groups do not necessarily turn people against a robot; group effects can also work in favor of robots in situations in which the group members support each other to explore the environment and a robot.

Family, friend, and female groups were highly entitative (H2ai, H2bii) and responded more positively toward the robot than others. These results support research indicating that different types of groups naturally have different levels of entitativity (Lickel et al., 2001). An alternative reason for increased interaction when in groups is because participants in groups could watch their families or friends interact with the robot, which accounted for some of the time and close interaction distance with the robot. However, this would not necessarily account for increased positivity of entitative groups. Robot designers may wish to target entitative groups of users to increase interaction time with their robots. A robot could initially target those in a group who are more likely to want to interact immediately with the robot (e.g., women in this study), and once the group members are present, could switch strategies to appeal to other group members. Indeed, once participants' groups were interacting with the robot, the participants themselves were more likely to interact with it.

### H3. Social Norms Affected Interaction

Participants followed group norms of interacting with the robot (H3). That is, when others in participants' group interacted with the robot, participants who did not typically interact were more likely to (H3a). Because males interacted less than females, as in previous observational research of naturalistic interactions with the robot in public spaces (Chang et al., 2014), this was especially pronounced for male participants (H3b). Further, once one's own group was interacting with the robot, males, and females were similarly likely to approach and interact. As suggested above, practitioners trying to increase interaction with the robot might initially target group members that are more likely to interact with the robot (females in this case), and once the group is interacting, engage other group members.

An alternate explanation is that female groups in the mall were more likely to be there for leisure whereas the male groups were more likely to be there for business. Therefore, those groups with more females were more likely to have the group purpose of exploring and therefore, have more positive interactions with the robot, whereas those in groups with more males were more likely to have the purpose of business and therefore, have fewer interactions with the robot. Regardless, these findings show that the group composition and purpose of the group bring participant behavior to be closer to the behavior of the other members of the group.

### Limitations, Design Recommendations, and Future Studies

In this study, we examined actual interaction with a robot in a real world setting, giving our results high external reliability. As such, none of the variables examined were experimentally manipulated, meaning that we cannot infer causation. Future studies should confirm that the effects we found can be replicated in more controlled studies during actual manipulation, and in other contexts. For example, in this study, it could be surmised that people at the mall for fun rather than business (i.e., family, friend, females, compared to males, coworkers) were more likely to interact with the robot, rate it positively, and interact with it for longer. This was especially true when they were in groups, especially more entitative groups. Future studies should examine if robots made for a work environment would attract more coworkers and if entitativity of coworker groups would also increase interaction time with the robot.

One confound in the study is that groups that are more entitative (family, friend, female) were also more likely to be in the mall for leisure than lower-entitative groups (coworkers, men). We recommend that scholars take these results with caution. Future studies should directly manipulate entitativity or examine entitative groups in different settings to disentangle these variables.

Further, family groups and female friend groups were not only more entitative, but perceived the robot as more positive than other groups. Future designers might market robots toward women and children for mall settings because these were the typical users in this study. However, the results indicate that it is important to remember that it is the social context (e.g., family outing) that is at least as important as gender in affecting responses toward a robot.

Additionally, this study was conducted in Japan. Findings, especially those related to business people and gender, may differ in different countries and in different social contexts.

In this study, we did not have enough survey data to examine the relationship between behavior toward the robot and survey ratings of the robot. Future studies could employ more surveys, such as by introducing a simple button near the robot to rate the interaction (positive, neutral, negative) for participants to employ after interaction to gain more explicit ratings of the robot.

Another limitation is that, although in some groups we were able to survey multiple participants, in other groups, only one participant would take the survey. This may bias the results if, for example, the person who was most likely to take the survey was the person who responded most positively to the robot. This type of self-selection bias is a limitation of all naturalistic studies that request survey participation. Examining these effects in a laboratory setting would circumnavigate this limitation. However, this is not a major concern because participant actual behavior supports conclusions drawn from surveys.

Finally, the video coders were not accurately able to identify family vs. friend groups. This could be a limitation in that we cannot make strong conclusions about behaviors of family and friends. However, if robots are programmed to identify different groups, they may also not be able to correctly identify family vs. friend groups. The identification of age-similar vs. age-diverse groups that were used in this study could plausibly be employed by robots in the near future, making this research directly applicable to today's HRI.

## Conclusion

Overall, in this study we sought to find how the presence of a groups, group characteristics, and group norms relate to people's behavior toward a humanoid mall guidance robot. The results indicated that, in this friendly context, groups, and especially entitative groups, were more positive toward a robot. Second, group norms of interacting with a robot influenced participants who would not normally interact with the robot to interact with the robot. Practitioners can apply these results to the design and implementation of public HRI, with robots targeting high-entitative groups if they are looking for longer interactions. Future studies might examine more ways to engage low-entitative groups and others that are less likely to interact with a public robot.

## Data Availability

The datasets generated for this study of survey and codes of video behavior are available through the Open Science Foundation (OSF) at https://osf.io/ew8ta/?view_only=df9ef0f0919b48afb64a773ffa0251ac.

## Ethics Statement

This study was carried out in accordance with the recommendations of Indiana University, Institutional Review Board with verbal informed consent from all survey subjects. The study was exempt from requiring written informed consent because the study was minimal risk and in a public domain. Video subjects were not required to provide written or verbal consent because they were in a public location; however, a large, prominent sign informed them that video recordings were being made in the location. The protocol was approved by the Indiana University Institutional Review Board.

## Author Contributions

The ideas for this paper were conceptualized and developed by MF, SŠ, and TK. MF ran the study, data analysis, and wrote paper drafts, MF consulted SŠ throughout the process of data collection, analysis, and paper writing. SŠ and TK significantly edited the paper.

## Contribution to the Field

Robots are increasingly being placed in public settings, including malls, museums, and city streets, to help guide and direct people. The results of this study demonstrate how the characteristics of human groups influence people's behavior when interacting with a robot. It presents the novel finding that groups (especially integrative groups), compared to individuals followed the group norm more for interacting with a robot. Researchers and practitioners can use this information when designing robots for public interaction with people, and for engaging people who might not otherwise be interested in interacting with a robot.

### Conflict of Interest Statement

The authors declare that the research was conducted in the absence of any commercial or financial relationships that could be construed as a potential conflict of interest.
