# Supplementary material for: Human Group Presence, Group Characteristics, and Group Norms Affect Human-Robot Interaction in Naturalistic Settings
Source: Front Robot AI. 2019 Jun 27;6:48. doi: 10.3389/frobt.2019.00048 (PMC7806044; doi:10.3389/frobt.2019.00048)
Supplement: Supplementary file 1 [file Presentation_1.pdf]

Appendix A

Survey in English

Please answer these questions about the robot.

1. Please rate this robot on the following traits:

Negative -------1-------2-------3-------4-------5-------6-------7-------Positive

Scary -------1-------2-------3-------4-------5-------6-------7------- Friendly

Mean -------1-------2-------3-------4-------5-------6-------7------- Kind

Useless-------1-------2-------3-------4-------5-------6-------7-------Useful

Stupid-------1-------2-------3-------4-------5-------6-------7-------Smart

Nonsocial-------1-------2-------3-------4-------5-------6-------7-------Social

Machine-like-------1-------2-------3-------4-------5-------6-------7-------Human-like

2. Did/would you enjoy interacting with this robot?

Definitely Not -------1-------2-------3-------4-------5-------6-------7-------Definitely

3. Would you use/interact with this robot in the future?

Definitely Not -------1-------2-------3-------4-------5-------6-------7-------Definitely

4. Would you recommend for others to use/interact with this robot?

Definitely Not-------1-------2-------3-------4-------5-------6-------7-------Definitely

5. How many people are in your group today? (Including you)

6. How would you describe the members of your group?

1 Family

2 Friends

3 Coworkers

4 Loose acquaintances

5 Do not know each other

6 I am alone

7 Other (please specify)

Please rate your agreement with the following statements. (1: Strongly disagree; 7: Strongly agree)

7. The group of people I am with today and I are a cohesive group.

8. The group of people I'm with today and I are similar to each other.

9. The robot and I are a cohesive group.

10. The robot and I are similar to each other.

11. What is your experience with computers?

    Novice/uncomfortable using computers

    Comfortable using computers for a few, simple tasks (e.g., write a note)

    Comfortable using computers for moderately complex tasks

    Comfortable programming computers

12. What is your experience with robots? (choose all that apply)

    None

    See robots in media (e.g., videos, TV)

    Interacted with robots (e.g., factory robots, roomba)

    Own 1+ robots

    Work with/build robots

13. Year born:

14. Gender:

4

Appendix B

Survey in Japanese

ロボットについてのアンケートにご協力ください。

1. 以下の項目で、ロボットを評価してください（該当する番号に丸をしてください）。

悪い-----１-----２-----３-----４-----５-----６-----７-----良い

恐い-----１-----２-----３-----４-----５-----６-----７-----心地良い

不親切-----１-----２-----３-----４-----５-----６-----７-----親切

不便-----１-----２-----３-----４-----５-----６-----７-----便利

馬鹿-----１-----２-----３-----４-----５-----６-----７-----賢い

非社交的-----１-----２-----３-----４-----５-----６-----７-----社交的

機械っぽい-----１-----２-----３-----４-----５-----６-----７-----人っぽい

2. このロボットとのやり取りは楽しかったですか？

全く思わない-----１-----２-----３-----４-----５-----６-----７-----そう思う

3. また、このロボットを使ってみたいと思いますか？

全く思わない-----１-----２-----３-----４-----５-----６-----７-----そう思う

4. このロボットの事を、友人・知人に薦めますか？

全く思わない-----１-----２-----３-----４-----５-----６-----７-----そう思う

5. 今日は何人で来られましたか？（ご自身も含めて）____________
6. どのようなご関係ですか？

家族　　友達　　同僚　　知人　　面識のない人　　一人　　その他
:_______________

7. 私たちのグループは、団結力のあるグループである。

強く否定-----１-----２-----３-----４-----５-----６-----７------強く同意

8. 私とグループのメンバーは、よく似ている。（趣味、仕事、興味、バックグラウンドとなる知識など）。

強く否定-----１-----２-----３-----４-----５-----６-----７------強く同意

9. 先ほどのロボットと私は、団結力のあるグループである。

強く否定-----１-----２-----３-----４-----５-----６-----７------強く同意

10. 先ほどのロボットと私は、お互いに似ている

強く否定-----１-----２-----３-----４-----５-----６-----７------強く同意

11. パソコンをどれくらい使えますか？

- ☐ 初心者/不慣れである。
- ☐ 簡単な作業は慣れている。（例：書類を書く）
- ☐ 適度に難しい作業を行うことには慣れている。
- ☐ プログラミングをすることに慣れている。

12. どのようにロボットと関わったことがありますか。（該当する項目の全てにチェックをお願いいたします。）

- ☐ 一度も無い
- ☐ メディアで見たことがある（動画、テレビなど）
- ☐ ロボットとインタラクションを取ったことがある。（例：工場内作業ロボット、ルンバ）
- ☐ １台以上のロボットを保有している。
- ☐ 一緒に働いている。/ロボットを製造している。

13. 年齢をお答え下さい。　______________________

14. 性別をお答え下さい。　_________________________
